# Early Tranexamic Acid Therapy and Its Influence on Hemoglobin Dynamics, Hospital Stay, and ICU Admissions in Upper Gastrointestinal Bleeding

**DOI:** 10.7759/cureus.100738

**Published:** 2026-01-04

**Authors:** Madina Riaz, Basil Usman, Muhammad Usama Talib, Jawaria Majeed, Sabahat Kiran, Samra Khalid, Muhammad Daud, Palwasha Abbasi, Muhammad Usman

**Affiliations:** 1 Acute Medicine, Queen Elizabeth Hospital Birmingham, Birmingham, GBR; 2 Internal Medicine, Isfandyar Bukhari District Hospital, Attock, PAK; 3 Orthopedic Surgery, University Hospitals of Morecambe Bay NHS Foundation Trust, Lancaster, GBR; 4 Gastroenterology and Hepatology, Nishtar Medical University - Multan, Multan, PAK; 5 Neurosciences, St. Martinus University Faculty of Medicine, Willemstad, CUW; 6 Internal Medicine, Shifa International Hospitals Limited, Islamabad, PAK; 7 Cancer Research, Rutgers Cancer Institute of New Jersey, New Brunswick, USA; 8 Gastroenterology, Rehman Medical Institute, Peshawar, PAK; 9 Pharmacology, Suleman Roshan Medical College, Tando Adam, PAK; 10 Medicine, Social Security Hospital, Sahiwal, PAK

**Keywords:** hemoglobin dynamics, hospital stay, icu admission, tranexamic acid, upper gastrointestinal bleeding

## Abstract

Background

Upper gastrointestinal bleeding (UGIB) is a life-threatening emergency associated with substantial morbidity, frequent transfusion needs, and prolonged hospitalization. Tranexamic acid (TXA) may reduce bleeding by stabilizing fibrin clots, but evidence regarding its early use in UGIB remains mixed.

Objective

This study evaluated whether early TXA administration influences hemoglobin dynamics, hospital stay, and ICU admissions in patients presenting with UGIB.

Methods

This prospective observational study was conducted at Shifa International Hospital, Islamabad, Pakistan, over 24 months. A total of 220 adult patients with UGIB were enrolled through purposive sampling and divided into two equal groups: an early TXA group receiving intravenous TXA within six hours of presentation (n=110) and a control group receiving standard care (n=110). Baseline demographic and clinical characteristics were similar between groups. Primary outcomes included hemoglobin levels at baseline, 24 hours, 48 hours, and discharge. Secondary outcomes included hospital length of stay, ICU admissions, blood transfusion requirements, and in-hospital mortality. Multivariable regression analyses were performed to adjust for age, comorbidities, baseline hemoglobin, bleeding source, and endoscopic interventions.

Results

Early TXA significantly preserved hemoglobin at 24 hours (9.2 ± 1.4 vs. 8.5 ± 1.6 g/dL, p < 0.001), 48 hours (8.9 ± 1.3 vs. 8.1 ± 1.5 g/dL, p < 0.001), and discharge (9.0 ± 1.2 vs. 8.2 ± 1.4 g/dL, p < 0.001). Hospital stay was shorter with TXA (5 (4-7) vs. 7 (5-9) days, p < 0.001), and ICU admissions were lower (10.9% vs. 23.6%, p = 0.008). TXA-treated patients required fewer transfusions (1.2 ± 0.8 vs. 2.0 ± 1.1 units, p < 0.001). Mortality was lower but not statistically significant (3.6% vs. 7.3%, p = 0.25). Multivariable analysis confirmed independent associations between early TXA and reduced hemoglobin drop, shorter hospital stay, and lower ICU admission odds.

Conclusion

Early TXA administration improves hemoglobin preservation, reduces transfusion needs, shortens hospitalization, and lowers ICU admissions in UGIB, supporting its role as an effective early adjunct in acute management.

## Introduction

Upper gastrointestinal bleeding (UGIB) remains a common and potentially life-threatening medical emergency associated with significant morbidity, mortality, and healthcare burden worldwide [1,2]. Despite advances in resuscitation, endoscopic techniques, and pharmacological therapies, early hemodynamic instability, recurrent bleeding, and transfusion requirements continue to challenge clinicians, often necessitating intensive care admission and prolonged hospitalization [3]. UGIB is estimated to affect 50-150 per 100,000 individuals annually, with mortality rates ranging from 5% to 10% despite modern interventions [2]. The severity of bleeding, rate of hemoglobin decline, and need for transfusion are critical determinants of clinical outcomes and resource utilization.

Tranexamic acid (TXA), a synthetic lysine analogue, is an antifibrinolytic agent that prevents clot degradation by inhibiting plasminogen activation [4]. Excessive fibrinolysis plays an important role in UGIB pathophysiology, particularly in peptic ulcer bleeding and portal hypertensive hemorrhage, where increased tissue plasminogen activator (tPA) and local fibrinolytic activity contribute to clot instability and recurrent bleeding [5,6]. By counteracting this mechanism, TXA may enhance clot stability, reduce ongoing bleeding, and diminish the need for transfusion and intensive monitoring.

Interest in TXA use for gastrointestinal bleeding dates back several decades, with early randomized trials showing reductions in rebleeding and transfusion requirements, though with variable effects on mortality [7,8]. More recent systematic reviews and meta-analyses have suggested potential benefits, including lower mortality, but with notable heterogeneity in dosing, timing, and patient populations [9,10]. Contemporary studies have emphasized the importance of early administration, drawing on strong evidence from large international trials in trauma (CRASH-2) [11] and postpartum hemorrhage (WOMAN) [12], both of which demonstrated substantial survival benefit when TXA was given promptly after bleeding onset. However, conflicting results from the HALT-IT trial, which employed prolonged high-dose infusion, raised concerns regarding safety and effectiveness in the context of gastrointestinal hemorrhage [13]. These discrepancies highlight the need to evaluate TXA specifically in early, short-course regimens that may offer hemostatic benefits while minimizing adverse events. The objective of this study was to evaluate the effect of early TXA on hemoglobin dynamics, hospital stay, and ICU admission rates in patients presenting with UGIB.

## Materials and methods

This prospective observational study was conducted at the Department of Gastroenterology and Emergency Medicine, Shifa International Hospital, Islamabad, Pakistan, over a period of 24 months, from January 2023 to December 2024. The study aimed to evaluate the influence of early TXA therapy on hemoglobin dynamics, hospital stay, and ICU admissions in patients presenting with UGIB. The study design and reporting were aligned with the Strengthening the Reporting of Observational Studies in Epidemiology (STROBE) guidelines to ensure methodological rigor and transparency. Patients were assigned to receive early TXA therapy based on attending physician discretion following informed consent; randomization was not performed due to ethical considerations and the emergent nature of UGIB. The sample size was calculated using OpenEpi software (Dean AG, Sullivan KM, Soe MM. OpenEpi: Open Source Epidemiologic Statistics for Public Health), based on a 95% confidence interval (CI), 80% study power, and an anticipated difference of 1 g/dL in mean hemoglobin drop between patients receiving early TXA therapy and those managed with standard care. The expected effect size was derived from prior observational studies on TXA in UGIB, particularly the HALT-IT trial, which reported clinically relevant reductions in blood loss among patients receiving early TXA administration [13]. Based on this calculation, a minimum of 200 patients was required, and to account for potential dropouts and incomplete data, a total of 220 patients were enrolled.

Patients were recruited using non-probability purposive sampling. This approach was chosen because it allowed the inclusion of all eligible patients presenting within the study period who met the specific clinical criteria for UGIB, ensuring feasibility within the busy hospital setting and allowing timely capture of early TXA administration. While purposive sampling is inherently non-random, the method was appropriate for this prospective observational study as the exposure (early TXA therapy) could not be assigned randomly due to ethical and logistical considerations.

Eligible patients were identified upon presentation to the emergency department or gastroenterology ward. Eligible patients had hematemesis, melena, or endoscopic evidence of bleeding. Patients presenting in overt shock (systolic BP <90 mmHg or requiring immediate ICU transfer) were excluded to prevent bias in ICU admission outcomes. Exclusion criteria included known hypersensitivity to TXA, pre-existing coagulation disorders, decompensated chronic liver disease (Child-Pugh Class C), ongoing anticoagulant therapy, pregnancy, recent thromboembolic events within the past six months, and patients who were transferred from other hospitals after initial treatment. All patients meeting the criteria were approached for informed consent, and enrollment continued consecutively until the desired sample size was achieved.

Data collection included detailed demographic information, comorbidities, medication history, hemodynamic status at presentation, baseline laboratory parameters including hemoglobin, and the source of bleeding as determined by endoscopy. Hemoglobin was measured using the Sysmex XN-1000 automated hematology analyzer (Sysmex Corporation, Kobe, Japan), employing the cyanmethemoglobin method with a precision of ±0.1 g/dL. Measurements were taken at baseline, 24 hours, 48 hours, and at discharge. Other outcomes included hospital stay, ICU admission, transfusion requirements, and in-hospital mortality. Outcome assessors were blinded to TXA timing to minimize bias. Early TXA therapy consisted of a loading dose of 1 g intravenously over 10 minutes, followed by 1 g IV every eight hours for 24 hours, in line with protocols used in previous UGIB studies. All other management, including proton pump inhibitors, endoscopic intervention, and etiology-specific therapy such as octreotide for variceal bleeding, was applied according to standard hospital protocols. Primary outcomes included hemoglobin dynamics measured at baseline, 24 hours, 48 hours, and at discharge, as well as hospital length of stay in days and admission to the intensive care unit. Secondary outcomes included blood transfusion requirements and in-hospital mortality. Potential confounders, such as age, baseline hemoglobin, comorbidities (e.g., diabetes, hypertension), source of bleeding, and concurrent therapies (e.g., proton pump inhibitors, endoscopic interventions), were recorded to allow adjustment in multivariable analysis.

Outcome assessors were blinded to the timing of TXA administration to minimize assessment bias. Patients were followed daily until discharge or death, and standardized hospital records were used to document laboratory values, interventions, and outcomes. To minimize loss to follow-up, only in-hospital outcomes were assessed, and all patients remained under continuous observation within the hospital system until discharge. Missing data, including dropouts or incomplete laboratory measurements, were addressed using multiple imputation for variables with less than 10% missing values. Variables with missingness greater than 10% were excluded from the analysis to avoid introducing bias.

Statistical analysis was performed using IBM SPSS Statistics for Windows, Version 26 (Released 2018; IBM Corp., Armonk, New York, United States). Continuous variables were expressed as mean ± standard deviation or median with interquartile range based on distribution normality assessed by the Shapiro-Wilk test. Categorical variables were presented as frequencies and percentages. Group comparisons between patients receiving early TXA and standard care were performed using independent t-tests or Mann-Whitney U tests for continuous variables and chi-square or Fisher’s exact tests for categorical variables. Multivariable linear and logistic regression models were used to evaluate the association between early TXA therapy and hemoglobin dynamics, hospital stay, and ICU admissions while adjusting for potential confounders. A p-value <0.05 was considered statistically significant. Sensitivity analyses were conducted in predefined subgroups based on age, baseline hemoglobin, and source of bleeding to assess the robustness of findings.

The study protocol was approved by the Ethical Review Board of Shifa International Hospital (Reference No. SIH/ERB/1167, dated: 14-10-2022), and written informed consent was obtained from all participants in accordance with the Declaration of Helsinki. All procedures, including laboratory assessments and endoscopic interventions, followed standard hospital protocols without requiring additional instruments or proprietary devices.

## Results

The study enrolled 220 patients with UGIB, evenly divided into the early TXA group and the control group. Baseline demographic and clinical characteristics, including age, sex, comorbidities, baseline hemoglobin, source of bleeding, and frequency of endoscopic interventions, were comparable between groups, with no statistically significant differences observed, indicating appropriate baseline comparability (Table 1).

**Table 1:** Baseline Demographic and Clinical Characteristics of Study Participants (n = 220) No significant differences were observed between groups, indicating baseline comparability. TXA: tranexamic acid; SD: standard deviation; χ^2^: chi-square test; t: independent t-test

Variable	Early TXA (n = 110)	Control (n = 110)	Test Statistic	p-value
Age (years, mean ± SD)	57.8 ± 12.4	58.3 ± 11.9	t = 0.31	0.76
Male, n (%)	68 (61.8)	65 (59.1)	χ^2^ = 0.21	0.65
Hypertension, n (%)	45 (40.9)	48 (43.6)	χ^2^ = 0.19	0.66
Diabetes mellitus, n (%)	32 (29.1)	30 (27.3)	χ^2^ = 0.10	0.75
Baseline hemoglobin (g/dL, mean ± SD)	9.8 ± 1.6	9.7 ± 1.5	t = 0.46	0.65
Source of bleeding (peptic ulcer), n (%)	56 (50.9)	54 (49.1)	χ^2^ = 0.06	0.81
Endoscopic intervention, n (%)	38 (34.5)	40 (36.4)	χ^2^ = 0.08	0.78

Analysis of hemoglobin dynamics demonstrated that patients receiving early TXA had significantly smaller reductions in hemoglobin at 24 hours, 48 hours, and at discharge compared to controls, while baseline hemoglobin was similar between groups (Table 2). This indicates that early TXA effectively preserved hemoglobin levels during hospitalization.

**Table 2:** Hemoglobin Dynamics in Early TXA Versus Control Groups Hemoglobin was measured at baseline, 24 hours, 48 hours, and discharge. Independent t-tests were used to compare groups. Early TXA significantly preserved hemoglobin levels. TXA: tranexamic acid; SD: standard deviation

Timepoint	Early TXA (g/dL, Mean ± SD)	Control (g/dL, Mean ± SD)	t-value	p-value
Baseline	9.8 ± 1.6	9.7 ± 1.5	0.46	0.65
24 hours	9.2 ± 1.4	8.5 ± 1.6	3.61	<0.001
48 hours	8.9 ± 1.3	8.1 ± 1.5	4.23	<0.001
Discharge	9.0 ± 1.2	8.2 ± 1.4	4.71	<0.001

Hospital stay was significantly shorter in the early TXA group, with a median of five days compared to seven days in the control group (p < 0.001). Additionally, ICU admissions were significantly lower in patients receiving early TXA (10.9% vs. 23.6%, p = 0.008) (Table 3), demonstrating a beneficial effect of early TXA on healthcare resource utilization.

**Table 3:** Hospital Stay and ICU Admissions in Early TXA Versus Control Groups Early TXA was associated with shorter hospitalization and fewer ICU admissions. ICU: intensive care unit; IQR: interquartile range; U: Mann-Whitney U test;  χ^2^: chi-square test; TXA: tranexamic acid

Outcome	Early TXA (n = 110)	Control (n = 110)	Test Statistic	p-value
Hospital stay (days, median (IQR))	5 (4-7)	7 (5-9)	U = 4212	<0.001
ICU admission, n (%)	12 (10.9)	26 (23.6)	χ^2^ = 7.12	0.008

Regarding transfusion requirements and mortality, patients in the early TXA group required significantly fewer blood transfusions (mean 1.2 vs. 2.0 units, p < 0.001). In-hospital mortality was lower in the early TXA group (3.6% vs. 7.3%), although this difference did not reach statistical significance (p = 0.25) (Table 4).

**Table 4:** Blood Transfusion Requirements and In-Hospital Mortality Early TXA reduced transfusion requirements, with a trend toward lower mortality. TXA: tranexamic acid; SD: standard deviation; t: independent t-test; χ^2^: chi-square test

Outcome	Early TXA (n = 110)	Control (n = 110)	Test Statistic	p-value
Blood transfusion (units, mean ± SD)	1.2 ± 0.8	2.0 ± 1.1	t = 6.23	<0.001
In-hospital mortality, n (%)	4 (3.6)	8 (7.3)	χ^2^ = 1.32	0.25

Multivariable regression analyses adjusting for age, baseline hemoglobin, comorbidities, source of bleeding, and endoscopic interventions confirmed that early TXA therapy was independently associated with a reduced hemoglobin drop at 48 hours (β = -0.68, 95% CI: -0.91 to -0.45, p < 0.001), shorter hospital stay (β = -1.5 days, 95% CI: -2.2 to -0.8, p < 0.001), and lower odds of ICU admission (adjusted OR = 0.41, 95% CI: 0.19-0.88, p = 0.02) (Table 5).

**Table 5:** Multivariable Analysis of Early TXA Effects on Clinical Outcomes Hemoglobin drop at 48 hours and length of hospital stay were analyzed using multivariable linear regression models and are presented as regression coefficients (β). ICU admission was analyzed using a multivariable logistic regression model and is presented as an OR. All models were adjusted for age, baseline hemoglobin, comorbidities, source of bleeding, and endoscopic intervention. OR: odds ratio; TXA: tranexamic acid

Outcome	Adjusted Measure	95% Confidence Interval	p-value
Hemoglobin drop at 48h (g/dL)	β = -0.68	-0.91 to -0.45	<0.001
Hospital stay (days)	β = -1.5	-2.2 to -0.8	<0.001
ICU admission	OR = 0.41	0.19 to 0.88	0.02

## Discussion

This study evaluated the impact of early TXA administration on clinical outcomes in patients presenting with UGIB. The findings demonstrate that early TXA significantly preserved hemoglobin levels at 24 hours, 48 hours, and discharge, reduced transfusion requirements, decreased ICU admissions, and shortened hospital stay. Because baseline demographic and clinical characteristics were comparable between groups, the observed differences are likely attributable to the early administration of TXA rather than confounding factors.

The significantly smaller decline in hemoglobin among patients receiving early TXA is consistent with the drug’s antifibrinolytic mechanism, which inhibits plasminogen activation and stabilizes fibrin clots, thereby reducing ongoing intraluminal fibrinolysis and microvascular bleeding [4,14]. The benefits observed in our cohort reflect findings from earlier randomized trials, which reported improved hemostasis and reduced active bleeding with TXA in peptic ulcer hemorrhage and other causes of UGIB [15-17]. Local fibrinolytic activity studies have demonstrated markedly elevated fibrinolysis at ulcer bases and variceal sites, providing a mechanistic rationale for TXA use in this setting [18]. The importance of early administration is supported by large-scale trials in trauma and postpartum hemorrhage, such as CRASH-2 [11] and WOMAN [12], which showed that TXA is most effective when given during the initial bleeding phase. Our results align with this principle, suggesting that early clot stabilization limits the extent of ongoing hemorrhage and improves hemodynamic stability.

The reduction in blood transfusion requirements in the early TXA group further supports the efficacy of early antifibrinolytic therapy. Previous trials have reported similar reductions in transfusion need, although findings across studies have been heterogeneous due to differences in dosing, timing, and patient selection [19-22]. By limiting early blood loss, TXA likely reduces the need for additional transfusions, which is particularly relevant in resource-limited settings and in patients at higher risk from multiple blood product exposures.

The significantly shorter hospital stay and lower ICU admission rates observed in the TXA group reflect improved early clinical stability and reduced severity of bleeding. Evidence from prior studies suggests that interventions enhancing early hemostasis can positively influence downstream outcomes such as hospitalization time and need for advanced monitoring [15]. Our findings suggest that early TXA administration reduces the progression of bleeding-related complications, thereby decreasing the likelihood of hemodynamic deterioration requiring ICU care. These trends mirror observations from trauma and obstetric literature, where early TXA administration consistently reduced progression to shock and organ dysfunction [14].

Although in-hospital mortality was lower in the TXA-treated group, the difference did not reach statistical significance. This is not unexpected, as our study was not powered to detect mortality differences, and mortality rates in modern UGIB management are relatively low. Earlier randomized trials also reported non-significant mortality reductions, but meta-analyses have suggested a modest overall survival benefit with TXA in UGIB [10]. Notably, our findings differ from those of the HALT-IT trial, which used a prolonged high-dose infusion and found no mortality benefit along with increased risks of venous thromboembolism and seizures [11]. The contrasting results likely stem from differences in dosing strategy, as shorter and earlier regimens, such as those in our study, may produce hemostatic benefits without the increased adverse event risk associated with prolonged high-dose exposure.

Early TXA administration may be a useful adjunct to standard UGIB management, particularly in settings where immediate endoscopy is unavailable or where blood product resources are limited. By preserving hemoglobin, reducing transfusion requirements, and improving hemodynamic stability, early TXA has the potential to reduce the intensity of care required and improve hospital resource utilization. However, the contrasting evidence from high-dose trials highlights the need for cautious patient selection and avoidance of extended high-dose infusions.

This study has notable strengths, including well-matched baseline characteristics, detailed measurement of hemoglobin trends at multiple time points, and the use of multivariable analyses to adjust for potential confounders. These factors strengthen the internal validity of the findings and offer robust evidence supporting early TXA use. However, certain limitations should be acknowledged. As a single-center observational study, residual confounding cannot be fully excluded despite statistical adjustment. The sample size, while adequate for evaluating intermediate outcomes, limits the ability to draw firm conclusions regarding mortality and rare adverse events such as thromboembolism. Another limitation is nonrandomization. Furthermore, the absence of routine screening for subclinical thrombotic events may underestimate the incidence of TXA-related complications.

## Conclusions

Early administration of TXA in patients with acute UGIB was associated with significantly better preservation of hemoglobin, reduced transfusion requirements, shorter hospitalization, and fewer ICU admissions, with a non-significant trend toward lower mortality. These findings support the use of early, short-course TXA as an adjunct to standard resuscitative and endoscopic therapy in UGIB. Further randomized controlled trials targeting early administration and optimized dosing strategies are warranted to confirm these benefits and clarify the safety profile in diverse patient populations.
